# Tolerability and efficacy of the intestinal phosphate binder Lantharenol^® ^in cats

**DOI:** 10.1186/1746-6148-8-14

**Published:** 2012-02-06

**Authors:** Bernard H Schmidt, Ute Dribusch, Peet C Delport, Jürgen M Gropp, F Josef van der Staay

**Affiliations:** 1 Bayer Animal Health GmbH, Global Drug Discovery & Development, Building 6700, 51368 Leverkusen, Germany; 2 ClinVet International Ltd, Bloemfontein, Republic of South Africa; 3 Faculty of Veterinary Medicine, University of Leipzig, Germany; 4 Emotion and Cognition Group, Farm Animal Health, University Utrecht, The Netherlands

## Abstract

**Background:**

Tolerability and efficacy of the intestinal phosphate binder Lantharenol^® ^(lanthanum carbonate octahydrate) were tested in two prospective, randomized and negative controlled laboratory studies with healthy adult cats fed commercial maintenance diets non-restricted in phosphorus. In the first study, the maximal tolerated dose was determined. Starting from a dose of 0.125 g/kg body weight mixed with the daily feed ration, the dose of Lantharenol^® ^was doubled every other week until signs of intolerability were observed (N = 10 cats compared to 5 untreated controls). In the second study, the effects of feed supplementation for two weeks with approximately 2, 6, and 20% of the maximal tolerated dose on phosphorus excretion patterns and balance were assessed (N = 8 cats per group).

**Results:**

Lantharenol^® ^was found to be safe and well tolerated up to the dose of 1 g/kg bodyweight, corresponding to a concentration of 84 g Lantharenol^®^/kg complete feed, defined as dry matter with a standard moisture content of 12%. Feed supplementation for two weeks with approximately 2-20% of this dosage (i.e., 1.6, 4.8, and 16 g/kg complete feed) resulted in a shift from urinary to faecal phosphorus excretion. Apparent phosphorus digestibility was dose-dependently reduced compared to the control group fed with diet only (N = 8).

**Conclusions:**

The feed additive was well accepted and tolerated by all cats. Therefore, Lantharenol^® ^presents a well tolerated and efficacious option to individually tailor restriction of dietary phosphorus as indicated, for instance, in feline chronic kidney disease.

## Background

Advances in veterinary medicine and animal nutrition have increased the life expectancy for companion animals [1]. This trend has been leading to an increase in the number of animals developing geriatric diseases, such as systemic organ disorders like chronic kidney disease (CKD). Further progress in medical and nutritional prevention as well as management of systemic organ disease is therefore required.

Although CKD and ensuing renal failure occur in cats of all ages, the prevalence of this disease increases with age [2]. CKD is diagnosed in 2-20% of all cats at some time of their life, and up to 31% of cats older than 15 years are affected [3]. In the majority of cases the underlying cause of CKD remains unknown. However, the resulting pathophysiology leads to a common endpoint of irreversible nephron damage. Cats may maintain a relatively stable state for a variable length of time. In most cases, CKD will progress to end-stage renal failure due to the self-perpetuating nature of the disease with an inherent progressive decline in renal function [4-6].

Distinct clinical signs and pathological changes in laboratory data are not usually present until more than 80-85% of all nephrons have lost their function [6]. At this stage, management of the CKD patient is aimed mainly at reducing clinical signs and slowing progression of the disease, if possible. Dietary modification is the mainstay of intervention [7,8]. It is generally designed to improve the quality and length of life, to assure an adequate intake of energy, and to minimize disturbances in fluid, electrolyte, vitamin, mineral, and acid-base balance [1,7]. It thereby can increase survival and decrease uremic crises, but in naturally occurring CKD it is not yet proven that disease progression itself is slowed by diet.

Although standard feline maintenance diets contain a high level of phosphorus (P) which is not a health risk in animals with normal renal function, retention and overload of P are inevitable consequences of CKD if intake of P is not restricted. Consequently, current guidelines for the management of CKD consistently underscore the importance of reducing P intake [5,7-10]. The restriction can be achieved by feeding a specially formulated diet, by adding an intestinal phosphate binder or by combining both approaches. Because protein sources of animal origin are particularly rich in digestible P, a low level of P in dietary formulations can only be achieved through restriction of protein. Unfortunately, diets with reduced protein content are often less palatable for cats, thus reducing compliance of animals to consume, and of their owners to feed the diets [8,11].

It is, however, of primary importance that cats with CKD maintain an adequate intake of nutrients and energy, and the restriction of dietary protein has to be carefully weighed against the risk of malnutrition [7]. Thus, P restriction with simultaneous maintenance of the standard protein level seems to be preferable in the early stages of CKD.

Phosphorus binding agents do not change dietary composition and supply, but rather decrease the gastrointestinal P absorption. Several calcium or aluminium based agents available for human use are described in protocols for the management of CKD in dogs and cats [2,8-10]. Although the risk and occurrence of side effects using these agents has been well documented for human patients, such evidence from studies conducted in companion animals is rare.

Lantharenol^® ^(lanthanum carbonate octahydrate) is a new intestinal P binder for animals. It is registered as a zootechnical feed additive by the European Commission [12]. The present article reports on its tolerability and efficacy in healthy cats fed standard feline maintenance diets non-restricted in phosphorus or protein.

Two independent studies were performed. In the first one, the dose of Lantharenol^® ^was escalated to identify the maximum dose that is accepted and tolerated by cats without causing any adverse effects. The objective of the second study was to establish a dose-effect relationship on P digestibility. Both studies were prospective, randomized, non-masked, negative-controlled laboratory studies.

## Results

### Study 1: Dose escalation study

Lantharenol^® ^administered in feed was well accepted by all of the cats up to a dose of 1 g/kg body weight. At this dose, only one cat left residuals of about 25% of its ration on two days out of the 2-week feeding period, yet still without signs of intolerability. Adverse effects were first observed when the amount of Lantharenol^® ^was increased to 2 g/kg body weight. The signs consisted of repeated vomiting of feed and occurred in seven out of the ten cats tested, starting on the second day of feeding the high dose (i.e., on study day 56), accompanied by refusal of test feed in three cats (see Table 1). Vomiting ceased after discontinuation of test item administration for two days (study days 59 and 60), but was observed again on the second day of re-challenge (study day 62), confirming the relationship of the adverse event to the test dose of Lantharenol^®^. No other signs of systemic or local gastrointestinal intolerability were observed in either treated or untreated cats.

**Table 1:** Dose acceptance and tolerability in cats subjected to a fortnightly dose escalation regimen of Lantharenol^® ^(Study 1)

Study group	Dose of Lantharenol^® ^(g/kg body weight)	Lantharenol^® ^content in feed original substance^1 ^(g/kg)	Lantharenol^® ^content in complete feed^2^ (g/kg)	Daily consumption of Lantharenol^® 3 ^(g/day)	Days of administration	No. of cats accepting/not accepting the feed	Signs of intolerability
A	0	0	0	0	63	5/0	None

B	0	0	0	0	14	10/0	None
B	0.125	2.7	10.2	0.4	14	10/0	None
B	0.250	5.4	20.5	0.8	14	10/0	None
B	0.500	10.8	41.8	1.5	14	10/0	None
B	1.000	22.1	84.4	3.1	14	9/1^4^	None
B	2.000	44.5	170.2	6.2	4^5^	7/3	Vomiting

The daily consumption, number of treatment days, number of cats accepting the feed and signs of intolerability observed were compared between a Lantharenol treated group (B; N = 10) and a negative control group (A; N = 5).

^1 ^IAMS adult, rich in lamb, 24% dry matter

^2 ^Complete feed is calculated by multiplying dry matter by the factor 1.12

^3 ^Group means

^4 ^One cat left 25% of its feed on 2 of 14 days

^5 ^The highest dose of Lantharenol^® ^was administered for 2 days, withdrawn for 2 days and then again given for 2 other days (re-challenge)

Apart from vomiting and reduced feed acceptance at the highest feed concentration of Lantharenol^®^, the general health status and characteristics of faeces were normal in all cats. During the physical examinations, no relevant signs of diseases were observed in any of the cats.

**Figure 1:**
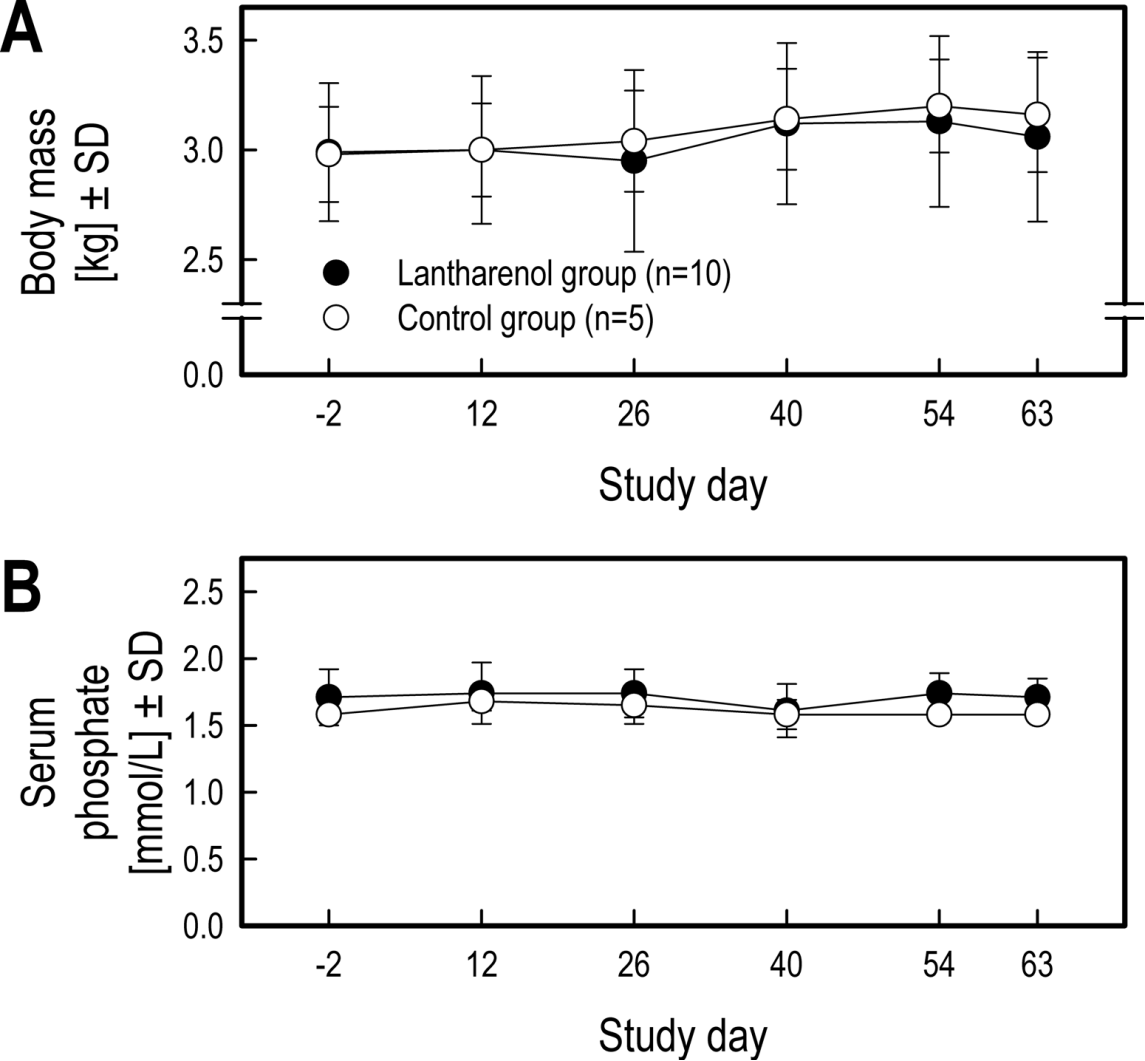
**Body mass and serum phosphate levels in cats subjected to a fortnightly dose escalation regimen of Lantharenol^® ^(Study 1)**. Panel A depicts the body mass (kg), serum phosphate levels (mmol/L) are shown in panel B as means and standard deviations of the Lantharenol^®^-treated and the untreated control group.

The average body mass during the study did not differ between groups (F_1,13 _= 0.07, NS, see Figure 1A). Over the course of the study, body mass slightly increased similarly in the two groups of cats (Study day: F_5,65 _= 5.43, p < 0.0003; Study day by Group interaction: F_5,65 _= 0.49, NS). The average serum phosphate levels were similar between the two groups of cats (F_1,13 _= 1.96, NS; see Figure 1B) and levels remained stable across all tested doses (Study day: F_5,65 _= 1.93, NS; Study day by Group interaction: F_5,65 _= 0.94, NS). Results are shown in Table 2.

**Table 2:** No effects of Lantharenol^® ^on P intake and P serum levels and difference of P serum level with respect to baseline in healthy adult cats (Study 2)

Measures	Dose of Lantharenol^® ^(g/kg feed original moist substance)			
	**0**	**0.3**	**1.0**	**3.0**

**P intake**				
Daily mean (mg)	200 ± 51	187 ± 51	230 ± 25	203 ± 48
**Serum P levels**				
Final (mmol/L)	1.28 ± 0.19	1.42 ± 0.23	1.32 ± 0.23	1.32 ± 0.17
Diff. to baseline (mmol/L)	-0.04 ± 0.09	0.04 ± 0.20	0.02 ± 0.09	-0.08 ± 0.20

Measures were taken on day -3 (baseline) and on day +14 and are depicted as group means ± standard deviation (N = 8 per group).

### Study 2: Dose-effect study

Feed supplementation with Lantharenol^® ^was well tolerated by all animals. No signs of decreased feed acceptance or other signs of intolerability were observed in any group. The results of clinical haematology and of blood chemistry at baseline and at the end of the study were within the reference range in all groups (data not shown).

Serum P levels remained stable and within reference ranges in all groups with no differences between control and treatment groups (F_3,28 _= 0.67, NS; see Table 2).

**Figure 2:**
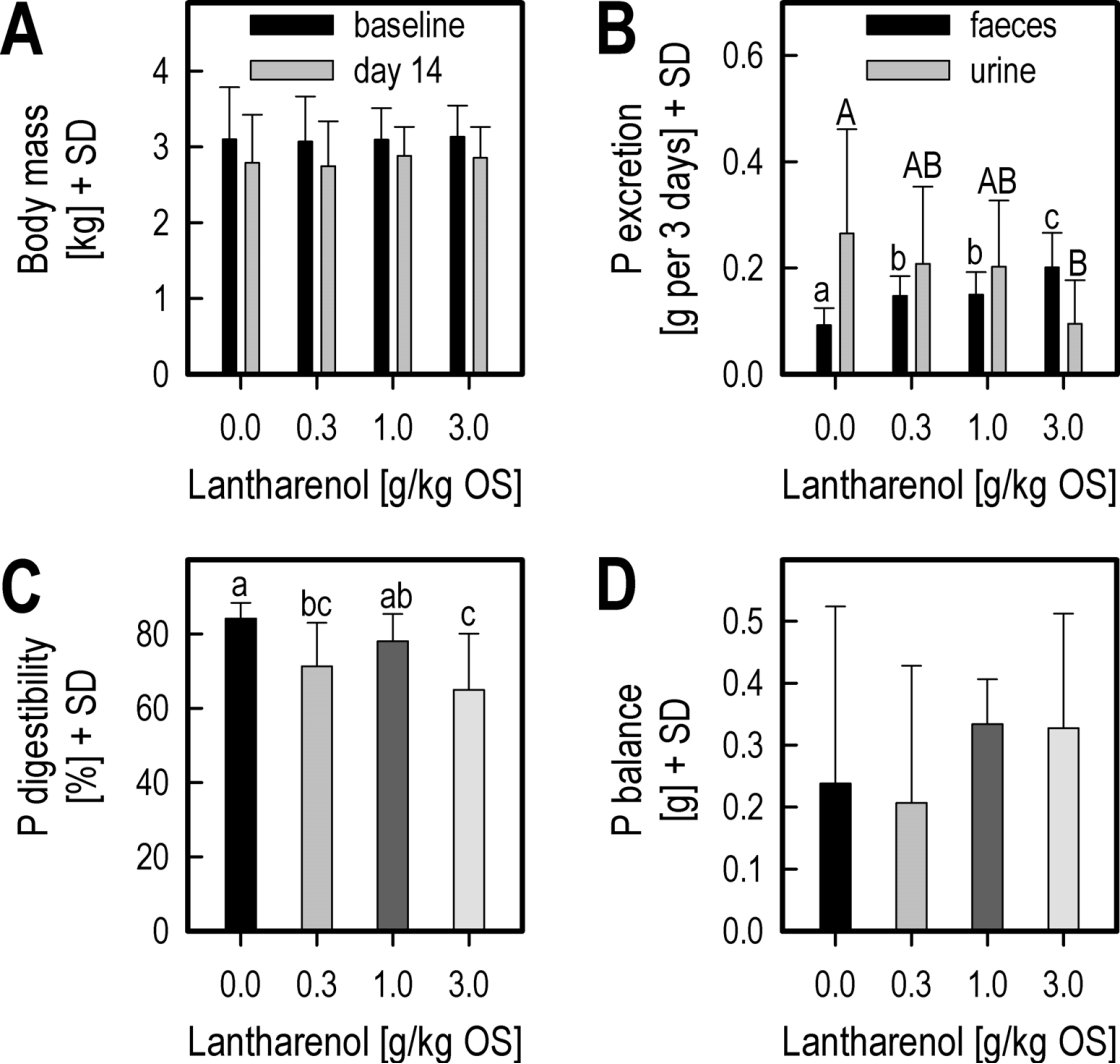
**Effects of feed supplemented with 0, 0.3, 1 or 3 grams Lantharenol per kg feed original moist substance in cats (Study 2)**. Body mass (kg) of four groups of cats fed cat feed supplemented with 0, 0.3, 1 or 3 grams Lantharenol per kg feed original moist substance (OS) on day -3 (baseline) and day 14 of the study (panel A), P excretion with urine and faeces (panel B), apparent P digestibility (panel C) and P balance (panel D) measured on day 14 of the study. The means and standard deviations are depicted per treatment group. Means with the same letter are not statistically different from one another (post-hoc tests). Note that the overall effect in urinary P excretion (panel B) was not significant.

The mean body mass decreased slightly from baseline (F_1,28 _= 239.53, p < 0.0001) and to a similar degree in all groups (Timepoint by Group interaction: F_3,28 _= 1.83, NS; see Figure 2A).

As can be expected with an intestinal P binder, P excretion in faeces dose-dependently increased (F_3,28 _= 20.43, p < 0.0001). This effect appeared to be accompanied by a corresponding decrease in urinary P excretion (see Figure 2B). This impression, however, was not confirmed statistically (F_3,28 _= 1.98, NS), whereas post-hoc comparisons pointed to a dose-dependent decrease.

Accordingly, mean apparent P digestibility was dose-dependently reduced by feed supplementation with Lantharenol^® ^(F_3,28 _= 5.04, p < 0.01; Figure 2C).

Phosphorus balance was not affected by the feeding Lantharenol-supplemented feed (F_3,28 _= 0.77, NS; Figure 2D).

## Discussion

The beneficial effects of dietary P restriction have been demonstrated in experimentally induced and in naturally occurring feline CKD. In cats with surgically reduced renal mass, dietary restriction of P intake markedly decreased renal pathology [13]. In a prospective study in cats with naturally occurring CKD, the mean survival time of the animals fed a veterinary diet restricted in P and protein was 2.4 times longer than that of cats maintained on a normal non-restricted maintenance diet. The beneficial effect was mainly attributable to P restriction [11]. In a retrospective study, Plantinga *et al. *[14] found similar effects on the survival time of cats fed different renal diets. A low level of dietary P was one of the main characteristics of the most effective diet.

In a prospective clinical trial, King *et al. *[15] found a highly significant negative correlation between plasma P concentration and survival time. The relevance of serum P in CKD was further confirmed in a retrospective study on predictors of survival time: Boyd *et al. *[16] found serum P concentration at time of diagnosis to be the only clinicopathologic variable tested that was predictive of survival.

Nutritional intervention to restrict P intake is considered as potentially effective to interrupt the vicious circle of inherent, progressive decline in renal function. In particular, the available evidence strongly supports the notion that this intervention is able to prevent renal secondary hyperparathyroidism and its sequelae [10,17,18]. A number of kidney friendly diets for cats and dogs specifically designed to restrict P intake are commercially available. They have a reduced content of protein and P and are adapted to further support nutritional needs of CKD patients. Restriction of protein is an important means to reduce P content because animal protein sources are intrinsically rich in P. However, the palatability of protein-restricted diets appears to be reduced, and consequently they are often less well accepted [5]. Dietary protein restriction must be carefully weighed against the risk of malnutrition and exacerbation of azotaemia, particularly in CKD patients where anorexia is already one of the most common signs.

Regular commercial maintenance diets for cats still remain relatively rich in P. In addition, the recommended daily requirement of P for adult cats appears to be overestimated [19]. This is particularly true for older animals with a reduced renal capacity for P clearance. With progression of CKD, the degree of P restriction needs to increase accordingly [8] which is difficult to achieve by mere restriction of P content in the feed through protein restriction.

As a potential alternative to dietary P restriction, or as an aid to further reduce the dietary burden of absorbable P beyond the technical and acceptance limits of restriction, an intestinal P binder can be added to the feed. Such agents bind diet-borne phosphate in the gastrointestinal tract and thereby reduce apparent P digestibility and systemic P availability.

In a recent review on the management of feline hyperphosphataemia, seven such P binding agents were listed: aluminium carbonate, aluminium hydroxide, aluminium oxide, calcium carbonate (with or without chitosan), calcium acetate, sevelamer hydrochloride, and lanthanum carbonate [10]. These compounds are derived from human treatment of renal hyperphosphataemia. With the exception of Lantharenol^® ^(lanthanum carbonate octahydrate), none of the P binders listed above has so far undergone any formal assessment by regulatory agencies for safety and efficacy as P binders for veterinary use, be it as a feed additive or as a pharmaceutical drug. Lanthanum carbonate tetrahydrate (Fosrenol^®^) is approved as a drug for treating hyperphosphataemia in human patients with end-stage CKD. Extensive trials proved that in this target population this P binder has few side-effects, which were mainly restricted to gastrointestinal tolerability [20]. It is an effective agent without significant risk of hypercalcaemia or worsening metabolic acidosis, and clinical studies in humans have demonstrated its tolerability for short- and long-term administration [21].

In the studies reported in the present paper, Lantharenol^® ^was found to be well tolerated and efficacious to reduce systemic P availability in healthy cats as well. It was well accepted and tolerated by healthy cats fed standard moist feline maintenance diets non-restricted in P and protein. General health status, P balance, feed acceptance, body weight changes and serum P levels were used as the main indicators for tolerability and systemic tolerability of Lantharenol^® ^in the range of feed concentrations tested, whereas the shift of P excretion from urinary to faecal excretion was used to assess efficacy.

Supplementation of feed with Lantharenol^® ^produced no adverse effects on feed acceptance and behaviour up to a dose of 1 g/kg body weight (corresponding to 84 g/kg complete feed). During a total of more than 800 individual feeding sessions in both studies, feed supplementation with Lantharenol^® ^was well accepted up to this maximal tolerated dose.

In the dose escalation study, vomiting first occurred at a dosage of 2 g/kg body weight in 7 out of 10 cats. As there had been an overall acceptance of the supplemented feed in all cats at dosages up to the maximal tolerated dose, it is assumed that acute gastrointestinal irritation and resulting gastrointestinal intolerability is the limiting factor for Lantharenol^® ^overdosing, consistent with findings reported in studies with Fosrenol^® ^in human patients [20].

General health status and serum P levels were unaffected within the total dose range of Lantharenol^® ^applied, including the maximal tolerated dose and beyond. Furthermore, there were no adverse effects on P balance, clinical haematology and blood chemistry values as determined in the dose-effect study.

In all groups including controls, body weights increased in the dose escalation study, and significantly decreased in the dose-effect study, even though the dosage of Lantharenol^® ^amounted to only 2-20% of the maximal tolerated dose determined and daily feed intakes were unchanged. The most likely explanation for the observed weight loss is the inevitably stressful individual housing during the three day urine and faeces sampling period. Another possible explanation is that the cats in the dose-effect study were not fed according to their actual needs. The energy density was not declared on the feed label, and ad libitum feeding was recommended by the manufacturer. To avoid potential conflicts with nutritional demands, the cats were switched to this feed about a month before the official acclimatisation period to ensure that they were given an appropriate daily ration during the study. In any case, an effect of the test item on body weight can be excluded, as all groups including the control group that did not receive Lantharenol^® ^were similarly affected, and feed intake was not affected by feed supplementation with Lantharenol^®^.

Lantharenol^® ^proved to be an effective P binder with a dose dependent effect. Dosages of 1.6, 4.8 and 16 g/kg complete feed resulted in a dose related increase in faecal P excretion. This increase appeared to be accompanied by a decrease in urinary P excretion, but statistical evaluation failed to support this impression. This failure is likely related to the large standard deviations for urinary P excretion. Part of this variability could potentially have been overcome by longer sampling times and/or within subject analysis of changes in P excretion patterns from baseline. However, the experimental design of the dose-effect study did not foresee a baseline phase and hence did not allow for a within subject analysis.

Feed supplementation with Lantharenol^® ^reduced mean apparent P digestibility by up to 22%. Slightly lower but significant effects were observed with the lowest dosage tested (-15% mean apparent P digestibility at 1.6 g/kg complete feed). The results of a study by Wagner *et al. *[22] showed a similar absolute reduction of P digestibility in a balance trial with healthy adult cats fed a commercial canned diet for senior cats supplemented with a formulation of calcium carbonate and chitosan. Positive effects of this formulation on serum P and parathyroid hormone levels in cats with surgically reduced renal mass were also described by Brown *et al. *[17]. However, until prospective data on the safety and tolerability of calcium-based P binders become publicly available, the risk of hypercalcaemia with vascular and soft tissue calcification should be considered before using such agents in nutritional management of CKD in cats [9,10,23]. In fact, where serum calcium concentrations are elevated, alternative calcium-free P binders are preferable.

Phosphorus binders based on aluminium have already been disfavoured for use in CKD because of the potential for development of aluminium toxicity [10,20,23]. Possible adverse side effects have not yet been systematically evaluated in companion animals, but recently, two cases of aluminium toxicity following administration of aluminium-based P binders have been reported in dogs [24].

Sevelamer hydrochloride (Renagel^®^), an organic polymer, is a relatively new P binder used in human patients on renal dialysis. In rats with experimentally induced CKD, sevelamer significantly inhibited the occurrence of hyperphosphataemia and protected against deterioration of renal function, although the mode of action for the protective effect remains unclear [25]. Its effects in companion animals have not yet been reported to our knowledge.

## Conclusions

The studies reported in the present paper show a statistically significant effect of Lantharenol^® ^on P excretion and apparent P digestibility from the lowest dosage tested (1.6 g/kg complete feed). Supplementation of regular maintenance feed with Lantharenol^® ^was well tolerated and accepted by cats up to the dose of 1 g/kg body weight, corresponding to a concentration of 22 g/kg feed original moist substance or 84 g/kg complete feed. These results indicate a safety margin of more than ten times the maximal feed concentration of 7.5 g Lantharenol^®^/kg complete feed approved for cats [12]. Further studies on Lantharenol^® ^including its efficacy in P-restricted diets and in cats with CKD, will be published separately.

## Methods

### Animals, housing and management (both studies)

All cats included in the studies were adult laboratory European shorthair cats. They were in good health status as determined by physical examination and biochemical and haematological blood values within the reference ranges. All cats were regularly vaccinated. The animals had been housed in the test facilities prior to start of the study. Therefore, the acclimation period before the first administration of the test item was limited to two weeks. In this period, the cats were familiarized with the study-specific diet and feeding conditions.

In both studies, the animals were maintained in environmentally controlled rooms. They were housed in groups and were confined to individual cages only during the daily four hours feeding period and for specimen collection. Drinking water was supplied *ad libitum*.

The general health status of the cats and signs of any untoward effects (e.g. changes in behaviour, adverse events), as well as individual feed intake and faecal characteristics, were monitored and documented daily throughout both studies.

The study protocols were designed in compliance with the national and international guidelines and laws concerning animal welfare. Regarding study 1, permission to use animals for experimentation was given by District Council Düsseldorf, Germany, file No. 50.5-240-1-37/01. Study 2 was ethically approved by Clinvet Animal Health Ethics Committee, Bloemfontein, Republic of South Africa. After study completion, all cats returned to the cat colonies of the test facilities for use in further studies, unrelated to the present ones.

### Study Procedures and Statistical Analyses

#### Study 1: Dose escalation study

A total of 15 adult (1- to 2-year-old) neutered female cats were randomly allocated to the study group receiving Lantharenol^® ^(10 cats) and to the control group fed the non-supplemented diet (5 cats).

The cats were fed a standard moist feline maintenance diet (IAMS Adult, Rich in Lamb, IAMS Pet Feed GmbH & Co, Schwalbach, Germany), containing 24% dry matter and 0.26% P in the original substance. The daily ration was adapted to the individual need as determined during the acclimation period. The amount of unconsumed feed was assessed after finishing the daily feeding period.

The test item was mixed daily into the individual feed portions of the Lantharenol^® ^group, starting with a dosage of 0.125 g/kg body weight on study day 0. The dosage was doubled every 14 days until signs of intolerability were observed. Cats in the control group were fed the same diet without any admixture for the entire duration of the study (see Table 1, first two columns).

Body weights were assessed weekly for tolerability observations and for calculation of the individual amount of test item before each dose escalation.

Blood samples for determination of serum P levels were collected under fasted conditions every 14 days (on study days -2, 12, 26, 40, and 54, i.e., 2 days before the next planned dose incrementation) and on day 63 (final examination). The samples were centrifuged at 4°C with 1800 rpm for 10 minutes and frozen. They were delivered within 3-4 hours for P determination according to standard procedures at a clinical laboratory (BIOFOCUS, Recklinghausen, Germany).

Repeated physical examinations were performed according to standard veterinary procedures before, during, and at the end of the study. Each animal was examined to assess the hair coat/skin, gingivae, respiratory tract, cardiovascular tract, digestive tract, urinary and reproductive system, musculosceletal system and the central nervous system.

Signs of intolerability and feed consumption were documented and reviewed clinically.

##### Statistical analysis

An analysis of the parametric data of systemic tolerability observations (body weight, serum P levels) was performed using the SAS^® ^statistical software package version 9.2 (SAS Institute, Cary, NC) for descriptive and exploratory statistical methods. Changes in body weight and serum P levels were analyzed by repeated measures ANOVA with the repeated measures factor 'Study day' and the fixed factor 'Group' (Lantharenol^® ^treated vs. untreated control). F-values with an associated probability < 0.05 were considered as statistically reliable.

#### Study 2: Dose-effect study

Thirty-two neutered adult (1-3 years old) cats of either gender were randomly assigned to 4 groups (A-D; N = 8 per group). All animals were fed a commercially available moist feline maintenance diet (Petley's Gourmet Supreme Beef Casserole; 16.9% dry matter, 0.19% P in original substance) once daily. The daily ration of feed was adapted to the individual need of the cats. The individual daily feed intake was quantified during the acclimation period (day -14 to day -1) and during the test item administration period (day 0 to +13).

Lantharenol^® ^was mixed into the individual daily feed portions to achieve concentrations of 0 (control, group A), 0.3 (group B), 1.0 (group C) and 3.0 (group D) g/kg feed original substance (corresponding to about 1.6, 4.8, and 16 g Lantharenol^® ^per kg complete feed, defined by the European Commission as 'air-dry' matter containing a standard moisture of 12%) over a period of two weeks. The dose range equated to approximately 2-20% of the maximal tolerated feed concentration determined in the dose escalation study.

The animals were monitored for potential signs of intolerability throughout the study. Body weights were determined before the start and at the end of the Lantharenol^® ^administration period (days -3 and +14, respectively).

Blood specimens were collected under fasted conditions on days -3 and +14. Cats were sedated to facilitate blood specimen collection (0.125 mL xylazine 2% per kg body weight intramuscularly; Bayer Animal Health GmbH). Specimens for clinical chemistry were centrifuged and dispatched in a cool box. Analyses were performed according to standard methods (haematology: ClinVet International Ltd., Bloemfontein, Republic of South Africa; clinical chemistry: PathCare Veterinary Laboratory, Belleville, Republic of South Africa). The samples were analysed to determine the standard full haemogram and clinical chemistry (including serum values of urea, creatinine, sodium, chloride, potassium, P, calcium, total serum protein, albumin globulin, liver enzymes, total bilirubin, amylase and glucose).

From day +11 through end of study on the morning of day +14, urine and faeces were collected quantitatively from the cat litter boxes. A non absorbent litter (Katkor, Rein Vet Products, The Netherlands; [26] was used for separate collection of urine and faeces. The three 24-hour-samples obtained from each individual cat during this period were pooled. Urine specimens were placed on ice and faecal samples were frozen at -20°C immediately after collection. Samples were then dispatched in a cool box for the determination of P content according to standard methods at clinical laboratories (urine: PathCare Veterinary Laboratory, Bellville, Republic of South Africa; faeces: ARC-ANPI Laboratory, Gauteng, Republic of South Africa).

From these values, as well as from the calculated dietary intake of P (daily feed intake in g × 0.19% P content in original feed), P balance and apparent digestibility of P in the feed were calculated with the formula:

$$\begin{array}{c} \text{P}\;\text{balance}\;\left(\text{g}\right)=\text{P}\;\text{intake}-\left(\text{P}\;\text{excretion}\;\text{with}\;\text{urine}+\text{P}\;\text{excretion}\;\text{with}\;\text{faeces}\right)\\ \text{Apparent}\;\text{digestibility}\;\text{of}\;\text{P}\left(\%\right)=\left[\left(\text{P}\;\text{intake}-\text{P}\;\text{excretion}\;\text{with}\;\text{faeces}\right)÷\text{P}\;\text{intake}\right]\times 100 \end{array}$$

Per dose of Lantharenol^® ^the individual data of feed acceptance, adverse events and/or changes in clinical state, haematology and blood chemistry were tabulated, and assessed for any values outside the reference ranges.

##### Statistical analysis

Test item dependent changes from baseline (day -3) to day +14 in body weight and serum P concentrations within each group and between the groups were analyzed statistically by repeated measures ANOVA with dose as fixed effect and time point as repeated measures factor. Dose effects on P balance, urinary and faecal P excretion were evaluated by an ANOVA with dosage of Lantharenol^® ^as the main effect. Post-hoc tests were used for between group comparisons. The level of statistical significance was set to p < 0.05.

## Competing interests

Bayer Animal Health has a financial interest in Lantharenol^®^. BHS and UD are employees of Bayer Animal Health. PCD received compensation as employee from ClinVet International Ltd for conducting the dose-effect study and reporting the results, and JMG received compensation as consultant to Bayer Animal Health GmbH. FJS declares that he has no competing interests.

## Authors' contributions

The experiments were conceived by BHS, UD, and JMG. They were fully funded by Bayer Animal Health and performed by the Contract Research Organisation ClinVet International Ltd, Bloemfontein, Republic of South Africa (PCD), and in the laboratories of the Sponsor company (UD). FJS analyzed the data. All authors contributed to, and approved the final manuscript.
